# Architecture and roles of periplasmic adaptor proteins in tripartite eﬄux assemblies

**DOI:** 10.3389/fmicb.2015.00513

**Published:** 2015-05-28

**Authors:** Martyn F. Symmons, Robert L. Marshall, Vassiliy N. Bavro

**Affiliations:** ^1^Department of Veterinary Medicine, University of CambridgeCambridge, UK; ^2^Institute of Microbiology and Infection, University of BirminghamBirmingham, UK

**Keywords:** periplasmic adaptor proteins, TolC, drug eﬄux pumps, type I secretion system, antibiotic resistance, membrane proteins, membrane transport, RND family pumps

## Abstract

Recent years have seen major advances in the structural understanding of the different components of tripartite eﬄux assemblies, which encompass the multidrug eﬄux (MDR) pumps and type I secretion systems. The majority of these investigations have focused on the role played by the inner membrane transporters and the outer membrane factor (OMF), leaving the third component of the system – the *Periplasmic Adaptor Proteins (PAPs)* – relatively understudied. Here we review the current state of knowledge of these versatile proteins which, far from being passive linkers between the OMF and the transporter, emerge as active architects of tripartite assemblies, and play diverse roles in the transport process. Recognition between the PAPs and OMFs is essential for pump assembly and function, and targeting this interaction may provide a novel avenue for combating multidrug resistance. With the recent advances elucidating the drug eﬄux and energetics of the tripartite assemblies, the understanding of the interaction between the OMFs and PAPs is the last piece remaining in the complete structure of the tripartite pump assembly puzzle.

## Introduction – Components of Tripartite Pump Assemblies and Specificity

Gram-negative bacteria have to export a number of cargoes across their double membrane, which presents a formidable barrier for free diffusion of molecules. Amongst a number of secretion systems (Gerlach and Hensel, 2007; Christie et al., 2014; Minamino, 2014; Nivaskumar and Francetic, 2014; Thomas et al., 2014; van Ulsen et al., 2014; Zoued et al., 2014), tripartite eﬄux assemblies have particular importance for multidrug resistance, a growing global problem (Silver, 2011; Piddock, 2012).

**FIGURE 1:**
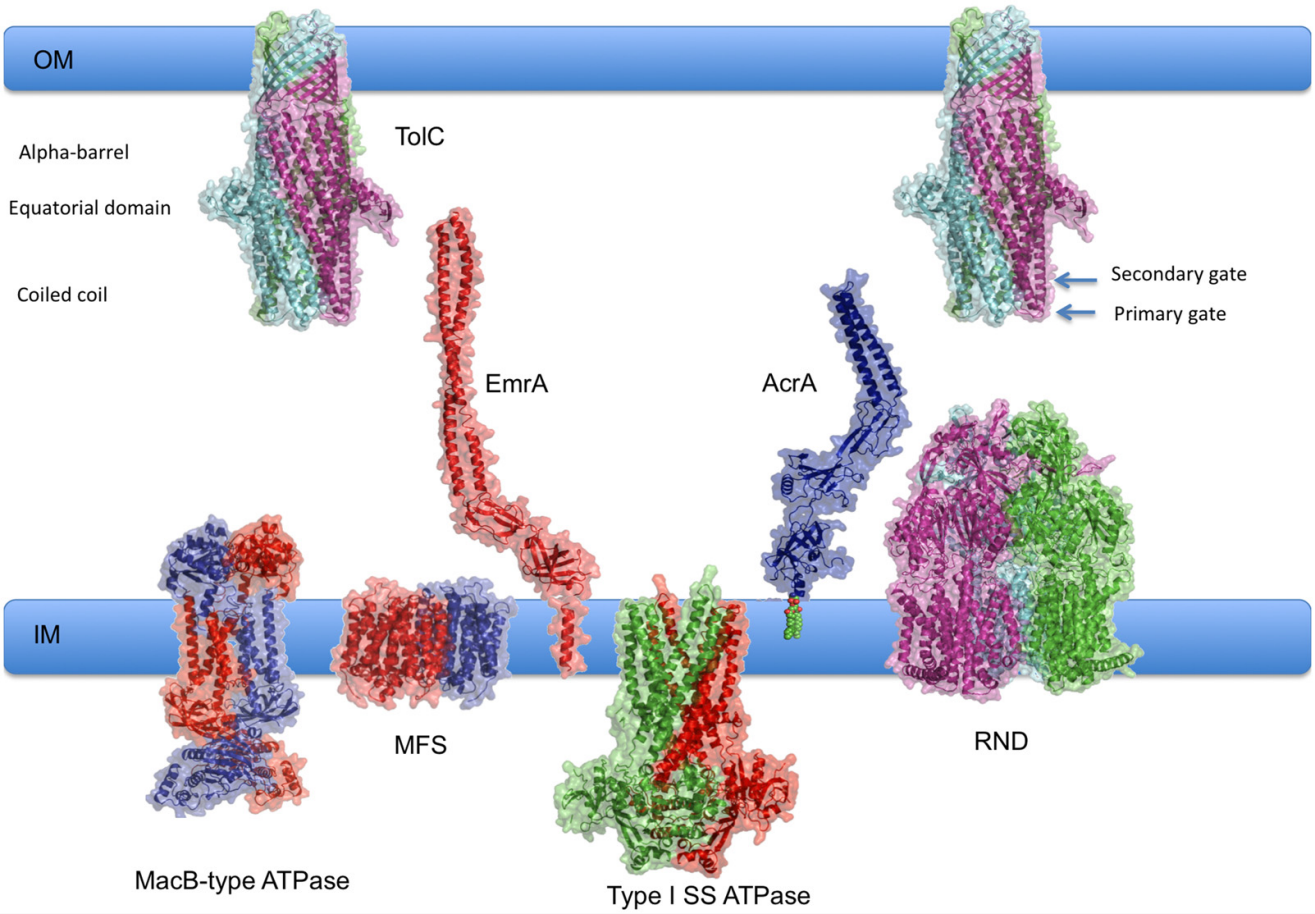
**Overview of tripartite assemblies engaged in eﬄux and type I secretion**. Schematic diagram of pump components showing their relative sizes and respective membrane locations. Representative experimental structures of RND transporter MtrD (4MT1.pdb); MFS transporter EmrD (2GFP.pdb); the OMF TolC (2VDD.pdb) and periplasmic adaptor protein (PAPs) EmrA (4TK0.pdb) have been used. Type I SS ATPase refers to ABC-transporters, such as HlyB, that are associated with Type I Secretion systems. Evaluative models of the components for which experimental structures are currently unavailable have been generated using homology modeling with I-TASSER (Yang et al., 2015) and manual optimisation using Coot (Emsley et al., 2010). The following templates were used: MacB (3FTJ.pdb); for HlyB (3ZUA.pdb; 2FF7.pdb; 2HYD), AcrA was modeled based on the experimental structure by Mikolosko et al. (2006) 2F1M.pdb. 3D structures in this manuscript were rendered using PyMol (The PyMOL Molecular Graphics System, Schrödinger, LLC.).

Tripartite assemblies are a heterogeneous group of *multidrug eﬄux* and *type I secretion systems* which draws from several different families of primary and secondary inner-membrane transporters (MFS, ABC and RND). With the help of the so-called *periplasmic adaptor proteins (PAPs)*, the inner-membrane transporters are linked to the *outer membrane factors (OMFs)* of the TolC family to create continuous conduits from the cytoplasm to the extracellular space, shown in Figure 1 (Misra and Bavro, 2009; Hinchliffe et al., 2013; Blair et al., 2014, 2015; Zgurskaya et al., 2015). These are involved in transport of cargoes that vary in size from single ions to large proteins, which could reach over 100 kDa (Kaur et al., 2012). In addition, a fourth transmembrane component is sometimes present in the complex, e.g., YajC (Törnroth-Horsefield et al., 2007) or AcrZ (Hobbs et al., 2012). These small proteins are entirely α-helical and bind the transporter within the inner membrane (Törnroth-Horsefield et al., 2007; Du et al., 2014). These proteins appear to be non-essential, but may play a modulatory role, affecting the eﬄux profile of the pump (Törnroth-Horsefield et al., 2007; Hobbs et al., 2012).

### Tripartite Eﬄux Assemblies and their Transporters

Multidrug eﬄux-pumps are grouped into a number of families including the primary transporters of the ABC-family [e.g., MacB (Kobayashi et al., 2003)], and secondary transporters which encompass the large group of RND-pumps (Eicher et al., 2014), major facilitator family (MFS), and a number of others, such as MATE, SM (Piddock, 2006; Bavro et al., 2008; Zgurskaya et al., 2015), and the recently discovered PACE family (Hassan et al., 2013, 2015). Of these, only the ABC, RND and MFS groups have been reported to participate in tripartite assemblies and associate with PAPs.

While the roles of the OMFs and transporters have been subject of much scrutiny (Koronakis et al., 2004; Zgurskaya et al., 2011; Ruggerone et al., 2013; Eicher et al., 2014; Wong et al., 2014; Du et al., 2015), the role of the PAPs has remained more obscure. Recent advances indicate that these diverse modular proteins, far from being passive linkers of the outer and inner membranes, are central players in the eﬄux and transport processes, including cargo recognition and selection, control of energy flow, and emerge as the main architects of the tripartite assemblies. As the phylogenetic connections of PAPs have been subject to thorough review (Zgurskaya et al., 2009), we will focus on summarizing the advances in structural knowledge of the PAP family and how it helps to better understand their function in the context of the complete pump assembly. Our analyses presented here indicate that adaptors possess a highly modular organization with structural domains shared beyond the adaptor protein group and re-used in a number of other protein components of transport and regulatory systems.

### The Outer Membrane Component – TolC

The OMFs, which are the outer membrane components of tripartite pumps, are trimeric integral membrane proteins. Although TolC was identified as a colicin-susceptibility factor in the early 1970s (Whitney, 1971), its association with multidrug eﬄux pumps was not conclusively proven until the mid-1990s (Fralick, 1996), when the whole family was described as membrane channels, or OMFs (Paulsen et al., 1997).

The structure of the prototypical member of the family, TolC, was solved by Koronakis et al. (2000) over a decade ago. Since then, the structural gallery has been expanded with the OprM (Akama et al., 2004; Phan et al., 2010); CusC (Kulathila et al., 2011; Lei et al., 2014a); VceC (Federici et al., 2005); MtrE (Lei et al., 2014b); and CmeC (Su et al., 2014). A detailed description of the structures of the OMF family is provided elsewhere (see Misra and Bavro, 2009; Hinchliffe et al., 2013) and a comprehensive review provides an overview of the functional characteristics of the family (Zgurskaya et al., 2011).

Outer membrane factors have a β-barrel domain resembling the porin fold, which, unlike the canonical porins is formed by all three subunits, each of which contributes four β-strands to form a pseudo-continuous barrel. In addition, OMFs possess a unique periplasmic domain, which, like the β-barrel, is a pseudo-continuous structure built with the participation of all three protomers. Unlike the β-barrel domain, the periplasmic part is almost entirely α-helical (Koronakis et al., 1997, 2000). The upper half of the periplasmic extension takes the form of an α-barrel domain (Calladine et al., 2001), while in the lower half this is an arrangement of coiled-coil hairpins – each subunit contributing two pairs of helices. This arises from the fact that each of the TolC protomers is itself a product of internal gene duplication, manifesting as a structural repeat, which effectively gives the TolC trimer a pseudo-sixfold symmetry. The overall β-barrel:α-barrel:coiled-coil architecture has been conserved in other TolC homologues crystallized since then, but some of the members, e.g., OprM, also present a flexible N-terminal tail, which is often lipidated and inserted in the outer membrane (Akama et al., 2004). Finally, in some OMFs the N-and C-terminal elements form an ‘equatorial domain’ about halfway up the periplasmic part of the protein.

In the original crystal structure of the TolC the coiled coils of the periplasmic domain curve inward below the level of the equatorial domain to give a closed pore extended into the periplasm (Koronakis et al., 2000). *In vitro* studies of the TolC channel in isolation showed that it is predominantly closed with only very short stochastic opening sequences, and exhibiting strong cation selectivity (Andersen et al., 2002b). The closure at the tip of the channel was revealed to be maintained by an elaborate network of charged interactions, involving D153, R367, Y362, which when disrupted resulted in leaky channel phenotypes (Andersen et al., 2002a; Augustus et al., 2004; Bavro et al., 2008). This network has also been analyzed by *in silico* molecular dynamics studies, which hinted toward the possibility of asymmetric channel opening (Schulz and Kleinekathöfer, 2009), and indicate that the channel may open more than seen in “open state” crystal structures (Bavro et al., 2008; Pei et al., 2011). Two aspartates of each monomer (D371 and D374), facing into the channel lumen at successive helical turns were identified as responsible for this cation selectivity (Andersen et al., 2002b). Similar constrictions are a common feature in the family and were observed in other members – including OprM and VceC (Akama et al., 2004; Federici et al., 2005). The nature of the selectivity gate may vary – such as in VceC, in which there is a hydrophobic constriction.

Bavro et al. (2008) suggested that the lower ion-bridges can be destabilized by direct interaction with transporters with large periplasmic domains, such as the RND family. The report also noted that the Asp-rings are too far up the channel to be directly affected by the transporter and are likely “unlocked” via interaction with the tip of the PAP. As successful unlocking of these bridges would be a requirement for productive transport, Bavro et al. (2008) designated them the “primary” and “secondary gates,” respectively. The designation indicates the sequence of cargo passage through these constrictions, although the order of their unlocking remains unclear.

### Determinants of OMF Specificity

While the adaptors and transporters are often encoded on the same operon, working in well-defined pairs that often stay associated even in the absence of substrate (Thanabalu et al., 1998; Zgurskaya and Nikaido, 2000), the outer membrane is served by only a handful of TolC-family members (Piddock, 2006; Zgurskaya et al., 2011). A consequence of this is that a number of different PAPs have to be able to bind to a single OMF, leading to “promiscuity” on the side of the OMF – in *Salmonella* at least 7 different eﬄux systems converge toward TolC (Horiyama et al., 2010). While a number of PAPs are able to function with a common OMF, this does not translate into total promiscuity; OMFs from one organism are not usually able to complement non-cognate systems. Even within one organism there is clear differentiation between systems on the basis of their OMF composition. As a result of this the focus of the search for determinants of specificity has justly fallen on to the OMF-adaptor interaction.

Periplasmic adaptor proteins successfully recognize and couple a limited set of OMFs to a diverse range of transporters, with high fidelity and selectivity of assembly. How they achieve this is one of the last remaining questions in the structure of eﬄux pumps. The answer has important medical implications due to the involvement of these complexes in both multidrug resistance and virulence (Nishino et al., 2006; Li and Nikaido, 2009; Nikaido and Pagès, 2012; Piddock, 2012, 2014).

## PAPs – Architecture and Structural Connections

### Discovery of the PAPs

The PAPs were initially identified as “membrane fusion proteins” (MFPs) based on perceived sequence similarity to *bona fide* viral MFPs, namely paramyxoviral SV5 fusion protein, and correspondingly a membrane fusion function was also ascribed (Dinh et al., 1994). The later description of the 3D structures of both MFPs and the paramyxoviral trimeric fusion protein (1ZTM.pdb) demonstrated a lack of general structural similarity between the two classes of proteins (Akama et al., 2004; Higgins et al., 2004b; Yin et al., 2005; Mikolosko et al., 2006). We show later that although viral MFPs and bacterial PAPs are generally dissimilar, one specific domain of the viral fusion protein structure can indeed be matched to a small domain in many PAPs. The inferred fusion function was never experimentally detected in the PAP family. Despite this, the term MFP has persisted, and can still be found widely in the literature. To avoid confusion we will use the alternative term “PAPs.”

### PAP Structures Solved to Date

The adaptor proteins were the last component of the tripartite pumps to be characterized structurally. In Akama et al. (2004) and Higgins et al. (2004b) the structure of the MexA from *Pseudomonas aeruginosa* became the first member of the family to be crystallized (1VF7.pdb and 1T5E.pdb), followed by the structure of AcrA (2F1M.pdb; Mikolosko et al., 2006). All of these structures were missing a significant region, later called the membrane proximal domain (MPD), which due to its highly flexible nature didn’t become available until re-refinement of the MexA structure by Symmons et al. (2009; 2V4D.pdb).

In quick succession, the MacA structures from *Escherichia coli* (3FPP.pdb) and *Actinobacillus actinomycetemcomitans* (4DK0.pdb) were solved (Yum et al., 2009; Xu et al., 2012), followed by a number of metal pump-associated PAPs – CusB alone (3H94.pdb; 3OOC.pdb; 3OPO.pdb; 3OW7.pdb; Su et al., 2009); ZneB from *Cupriavidus metallidurans* (3LLN.pdb; De Angelis et al., 2010); as well as the CusBA complex (4DNR.pdb; 3T51.pdb; 3T53.pdb;3T56.pdb; 3NE5.pdb; 4DNT.pdb; 4DOP.pdb; Su et al., 2011, 2012). The partial structure of the *Campylobacter jejuni* AcrA in a glycosylated state has also been determined by NMR (2K32.pdb; 2K33.pdb; Slynko et al., 2009). In addition, the structure of the PAP (BACEGG_01895) from a putative eﬄux pump from *Bacteroides eggerthii* DSM 20697 (4L8J.pdb) has become available from a structural genomics effort. Last year saw the report of the first MFS-transporter associated PAP – EmrA from *Aquifex aeolicus* (Hinchliffe et al., 2014), as well as a non-typical PAP lacking the α-hairpin domain, BesA (Greene et al., 2013), widening our picture of structural diversity of the family.

There are now example structures available of PAPs from RND systems, both small molecules and metals, and ABC-eﬄux systems, but to date no structure of a PAP from a Type I system.

### General Architecture and Domain Organization of PAPs

**FIGURE 2:**
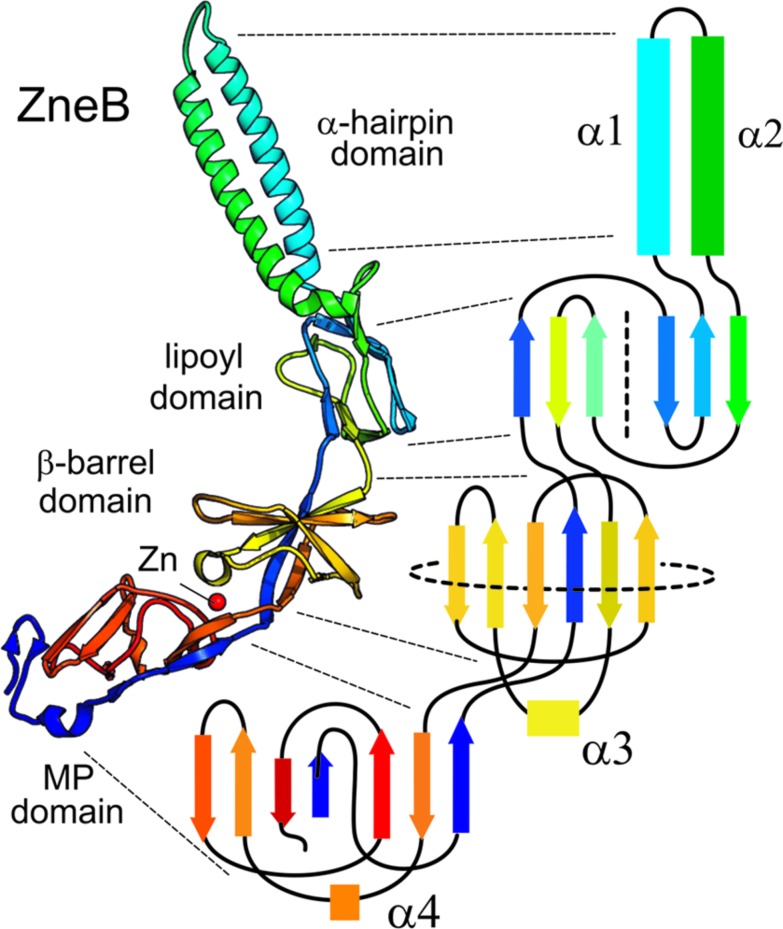
**Complete topology of a typical PAP**. The metal eﬄux adaptor ZneB is shown here in schematic form **(left)** colored from blue (N-terminal) through red (C-terminal). The overall topology is presented alongside **(right)** in equivalent colors for the β-strands and α-helices of each of the domains. The lipoyl domain has been flattened into two halves separated by a dotted line; and the β-barrel domain has also been flattened out as indicated by the circular dotted line.

Adaptor proteins are elongated molecules composed of a number of well-defined structural modules. Some modules are universal while others are only shared within a subset of the family. PAP structures show a ‘hairpin like’ arrangement in which the polypeptide passes from the inner-membrane outward to contact the outer membrane component and then back to the inner membrane (Figure 2). A topological analysis of domains in a complete adaptor (Figure 2, which has ZneB as an example) clearly shows how each domain is constructed from structural elements from the N- and C-terminal halves of the protein.

The central section of the majority of solved adaptors is an α-helical hairpin forming a coiled-coil arrangement. This is of variable length and in the PAP of one system (BesA) it is dispensed with entirely (Greene et al., 2013). The coiled-coil is extended and shortened by insertion or deletion of heptad repeats in the two α-helices. In the case of the metal eﬄux adaptor CusB, the hairpin is observed to be folded back on itself to generate a shortened four helical bundle (Su et al., 2009). In some PAPs the α-hairpin is extended by a further α-helical section constructed from paired α-helices. Similar to the helices in the TolC α-barrel, these run anti-parallel but without the marked twist of the coiled-coil helices. Crystal contacts in several PAP structures produce a six-membered barrel from these pairs of helices (see Yum et al., 2009, for example). This was suggested to function as a periplasmic channel assembly complementing the TolC periplasmic tunnel, based on similarity of their diameters although definitive evidence is not yet available.

Adjacent to the hairpin and its helical extension is a domain that was predicted and subsequently shown structurally to be homologous to biotinyl/lipoyl carrier domains in dehydrogenase enzymes (Johnson and Church, 1999; Higgins et al., 2004a). These domains consist of a β-sandwich of two interlocking motifs of four β-strands (Figure 2). Strikingly the α-hairpin is an extension from the same loop in this domain that contains the lysine which is modified with the lipoyl group in the dehydrogenase subunit. However, the PAP lipoyl domain does not contain the signature modified lysine, as the hairpin extension is spliced *en lieu* of the loop that harbors it. While the exact functional role of this domain is still to be established, analysis of mutations targeting it suggest that it has a role in stabilizing the complex assembly. This may be achieved either by interaction with the transporter, as indicated by cross-linking of the AcrA lipoyl domain to AcrB (e.g., Symmons et al., 2009), or by self-association, which would explain the loss of hexamerization of DevB when its lipoyl domain is disrupted (Staron et al., 2014).

The next domain in PAPs is a β-barrel consisting of six antiparallel β-strands capped by a single α-helix. The overall topology of this barrel (Figure 2 presents a limited 2D depiction) is also similar to enzyme ligand-binding domains such as the flavin adenine nucleotide-binding domain of flavodoxin reductase and ribokinase enzymes, and also to domains with odorant-binding properties (Higgins et al., 2004a).

A fourth domain present in some PAPs is the MPD (Symmons et al., 2009). Even when present, this is often ill-defined owing to its highly flexible connection to the β-barrel. Although it is constructed largely from the C-terminal elements of the protein, and has been termed ‘C-terminal domain,’ it also incorporates the N-terminal β-strand, which provides the direct link to the inner membrane. The first example of a MPD structure was revealed only after re-refinement of MexA crystal data, showing a β-roll that is topologically related to the adjacent β-barrel domain, suggesting that it is likely to be the result of a domain duplication event.

Periplasmic adaptor proteins are anchored to the inner membrane either by an N-terminal transmembrane helix or, when no transmembrane helix is present, by N-terminal cysteine lipidation (e.g., triacylation or palmitoylation) following processing by signal peptidase 2.

Periplasmic adaptor proteins associated with the heavy metal eﬄux (HME) family of RND transporters may also present additional N- and C-terminal domains. Involvement of the latter in metal-chaperoning function has been demonstrated in the SilB adaptor protein from *Cupriavidus metallidurans* CH34 (Bersch et al., 2011). These domains also present themselves as stand-alone proteins (e.g., CusF of *E. coli*) and possess a unique metal-binding β-barrel fold (Loftin et al., 2005; Xue et al., 2008). The domain of the SilB metal-eﬄux adaptor has been solved separately from the full length SilB adaptor.

**FIGURE 3:**
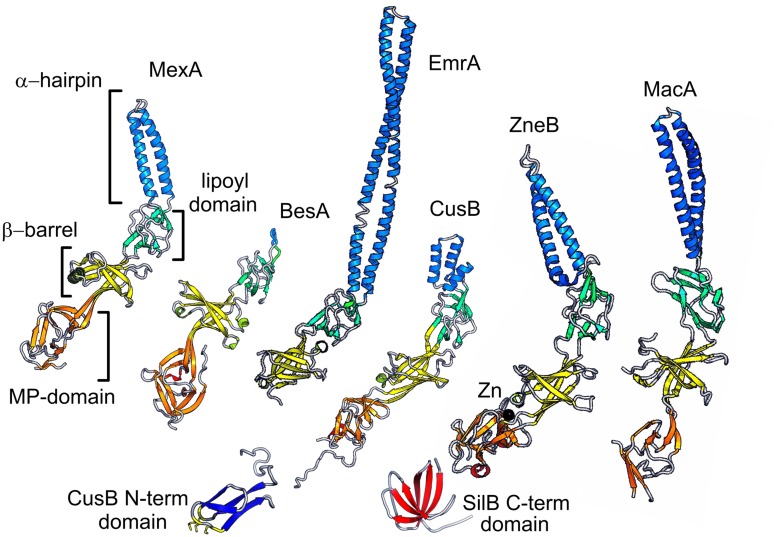
**Representative PAPs**. Selected examples of the PAP family are shown in schematic representation. The domains of MexA (RND adaptor) are indicated and colored orange for the MP domain, yellow for the barrel domain, green for the lipoyl, and blue for the hairpin. The equivalent domains in other examples are colored similarly. BesA (RND), which lacks the hairpin domain, EmrA (an MFS adaptor) which does not have an MP domain. CusB and ZneB are metal RND eﬄux pump adaptors some of which have additional domains represented here: the CusB N-terminal domain; and the SilB C-terminal domain. Finally the MacA ABC adaptor is shown.

The possible conformational transitions associated with ion binding in CusB have recently been revealed by modeling of the N-terminal domains based on extensive homology modeling combined with molecular dynamics and NMR spectroscopy data (Ucisik et al., 2013). Despite these advances there is limited structural data on the N-terminal domains at present. However, the CusB N-terminal domain can be modeled as shown in Figure 3 with the methionine residues implicated in metal binding clustered at one end of the domain.

### Structural Similarities Suggest Domain Duplications

**FIGURE 4:**
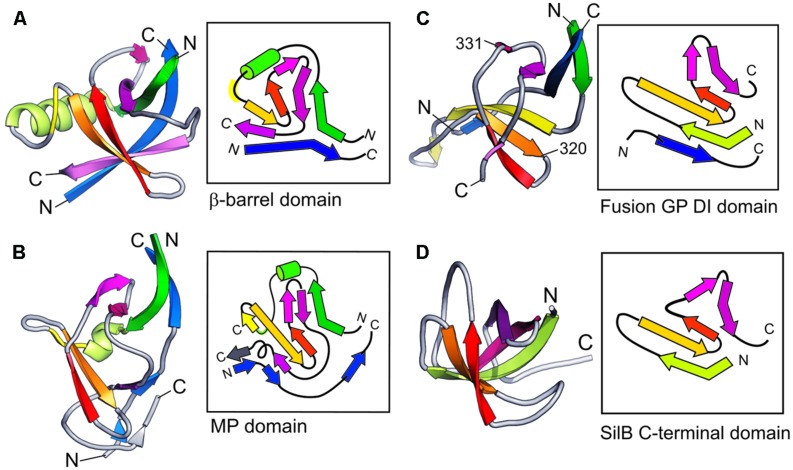
**Topological organization of PAP domains**. Side-by-side comparison of typical adaptor domains compared as 3D schematics colored from N- to C-terminal together with highly simplified topological diagrams in the same colors. **(A)** MexA β-barrel domain. **(B)** MexA MP domain. **(C)** Viral Fusion Glycoprotein DI domain (from 2B9B.pdb). **(D)** SilB C-terminal domain.

**Figures 4A,B** show the comparison of the detailed topology of the β-barrel and the MPDs from MexA. The key conserved elements in these domains is the combination of a β strand with a helix or helical turn (shown in green) followed by a β-meander (yellow, orange, red). The subsequent β-hairpin strands (magenta, purple) and an N-terminal strand (blue) are associated with this β-meander in the complete barrel domain. In contrast the MPD has a split in the barrel giving a β-roll structure. There is a characteristic folding over of the β-hairpin (Figure 4B, magenta, purple) and the N-terminal strand (blue) is also split so that it interacts with both halves of the MP domain.

Strikingly this combination of a β-meander with a β-hairpin is also observed in domain I of a viral fusion glycoprotein (Figure 4C, Fusion GP DI domain, from 2B9B.pdb) although the helix has been lost in this case. The resemblance is increased by the fact that the viral domain also shares the involvement of a separate, more N-terminal, strand. It is not clear if this structural similarity is in fact owing to evolutionary homology. However, it is possible that this structural resemblance underpins the original sequence similarity that motivated the name MFP for the adaptor protein family.

There is also a distant resemblance between the barrel/MP domain and the CusF metallochaperone topologies. This is shown in Figure 4D. Again there is no helical element and further there is no involvement of an N-terminal strand. Instead the barrel is completed by the β-hairpin (magenta, purple) folding back over the β-meander. The resulting topology is known from many OB-fold domains (Murzin, 1993), but may possibly have arisen as a special example of that fold in the case of the metal-eﬄux adaptors.

### Flexible Linkers in Periplasmic Adaptor Protein Structure

Owing to the hairpin-like pathway of the polypeptide chain through the PAP structure the linkers between each domain consist of two anti-parallel strands or turns. These are flexible but have distinctive structures with some degree of inter-strand hydrogen bonding. Comparing different PAP structures and also separate examples from different crystal environments shows these linkers can accommodate a range of both angular and rotational flexibility between adjacent domains.

These linkers are likely to allow the domains to optimize their individual interactions both with each other and with the inner and outer membrane components. This may be of significance as the TolC outer membrane exit duct undergoes conformational change on opening while the inner membrane transporter can undergo conformational changes as part of its pumping cycle. The associated PAPs must accommodate these conformational changes while retaining contact with the other pump components.

### Structural Homology and Evolutionary Connections of Periplasmic Adaptor Protein Domains

**FIGURE 5:**
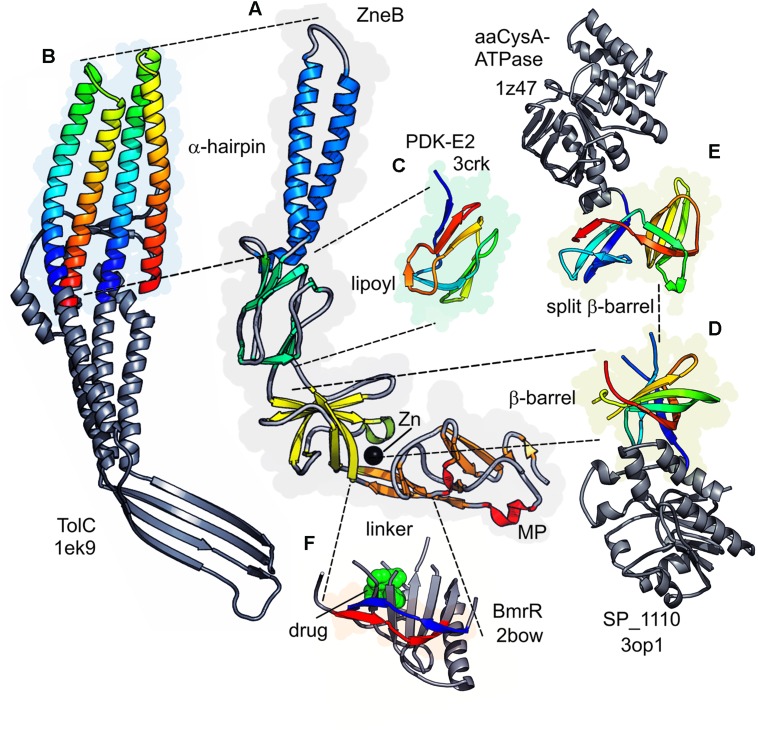
**Structural similarities with PAP domains**. The representative Adaptor ZneB is shown in the center **(A)** with domains colored as in Figure 3. Equivalent domains in other proteins are connected by dotted lines with their individual elements colored blue to red (N- to C-termini) and spacefilling envelop colored in the domain color. None equivalent domains are shown in gray. **(B)** The TolC subunit (1EK9.pdb) inverted to show the match between the two coiled coils and the helical hairpin. **(C)** The PDK-E2 subunit (3CRK.pdb) lipoyl domain. **(D)** The ribokinase-type barrel from the *Streptococcus pneumoniae* macrolide-eﬄux transporter SP_1110 (3OP1.pdb). **(E)** A modified split barrel from CysA ATPase subunit of the ABC transporter from *Alicyclobacillus acidocaldarius* (1Z47.pdb). **(F)** Relationship between the linker region between the β-barrel and MPD of the PAPs and the BmrR transcriptional regulator.

Periplasmic adaptor protein structures revealed that they have a common modular architecture. Far from being unique, their domains and linkers appear to be shared with other, highly diverse protein families, some of which are involved in bacterial tripartite systems and their regulation. Suggested structural relations of the adaptor domains to other proteins are shown in Figure 5.

It has been previously observed that the α-helical domains of particular PAPs resemble inverted versions of the TolC domains (Symmons et al., 2009). Strikingly the polypeptide also follows a similar pathway outward and back through each domain. The backbone of the α-helical hairpin domain from PAPs can be superimposed on both coiled-coils of TolC (Figure 5B) when inverted and viewed in an equivalent orientation. Furthermore, the α-helical hairpin extension domain of adaptors such as EmrA (Figure 3) and MacA is highly similar to the untwisted pairs of α-helices in the TolC α-barrel. Indeed MacA and related PAPs are observed to form a barrel-like hexameric assembly that superimposes very well on the complete lower part of the TolC trimer.

The lipoyl domain of PAPs was named owing to its sequence homology to the section of dehydrogenase enzymes (Johnson and Church, 1999). This homology was confirmed by the structure of MexA and subsequent adaptors showing that this region of the PAPs is topologically equivalent to those in lipoyl domains of dehydrogenases. This is clear when they are presented side-by-side in matching orientations, e.g., alongside the pyruvate dehydrogenase kinase (Figure 5C).

The β-barrel domain, adjacent to the lipoyl in the PAP structure, shares the topology of a barrel in ribokinase enzymes and lipid-binding proteins (Higgins et al., 2004b). It is intriguing that in one case such ribokinase-like barrel domain is also associated with a macrolide eﬄux protein of a Gram-positive organism – SP_1110 from *Streptococcus pneumoniae* (pdb structure 3OP1, compared with the adaptor in Figure 5D). A splitting of this barrel is observed in the cytoplasmic regulatory domain of another structurally characterized ABC transporter system – namely the sulfate transporter from *Alicyclobacillus acidocaldarius* CysA (Figure 5E). There, a partial duplication and rearrangement of the barrel strands in the CysA subunit may be recapitulating the changes in adaptor domain from a barrel to an MPD (Scheffel et al., 2005, 1Z47.pdb, Figure 5E).

These ribokinase-like domains are present in ABC-ATPases of the CUT1 and MOI subfamilies (Diederichs et al., 2000), which have been suggested to be involved in regulatory processes. Furthermore there is some evidence that these domains may play a role in signal transduction (Scheffel et al., 2005). Sequence alignments indicate (data not shown) that there is a high probability of a similar fold existing in MacB-type ATPases. While the evolutionary connection between these ABC-transporter associated domains and the β-barrel domain in PAPs remain to be fully established, the structural match is rather striking and would be consistent with the modular re-use of structures in these systems.

It is notable, that ribokinase-like domains reappear in some flagellar basal body assembly proteins (see Supplementary Figure S1). The C-domain of the flagellar protein FlgT from *Vibrio* (3W1E.pdb; Terashima et al., 2013), the role of which is not completely clear, but which has a remarkable structural connection to the N-terminal domain of the β-subunit of F1-ATPase, the catalytic subunit of the ATP synthase complex. Despite lacking a discernible sequence homology, the FlgT exhibits the same topology as the PAP β-barrel domains and is comprised of six β-strands forming a barrel, topped with a helix (see Supplementary Figure S1A). Interestingly, FlgA, a different flagellar P-ring associated protein, displays a topologically different, but structurally equivallent domain (3TEE.pdb; Supplementary Figure S1B), which, however, lacks a full complement of β-strands, leaving it incomplete.

Another example of possible structural re-use is provided by the extended linker between the barrel domain and the MPD, in those PAPs which have the latter feature. This linker, although an apparently simple arrangement of two antiparallel β-strands, provides conformational adaptability to allow the flexible arrangement of the barrel and MPD relative to each other. This has been suggested to help maintain association with the inner membrane transporter domains during pumping activity (Symmons et al., 2009). Intriguingly, however, a very similar extended linker connects the two halves of the intracellular regulatory domain from the transcriptional repressor protein BmrR in *Bacillus* (Figure 5F, 2BOW.pdb, Zheleznova et al., 1999). The BmrR repressor regulates the expression of a drug eﬄux system (Kumar et al., 2013), and the domain containing the ‘linker’ element is implicated in drug sensing (bound drug shown as spacefilling atoms, Figure 5F). It may therefore be possible that the linker element may have been reused during evolution of the regulatory system.

One final overall structural similarity which is difficult to ignore, is between the overall architecture of PAP assemblies and the packing of the domains of flagellin to give flagella assemblies (Yonekura et al., 2003). Although the detailed topology and connectivity differs from that of PAPs (Figure 2), the overall arrangement of a central paired helices surrounded by small β-stranded domains is similar. In the case of flagellin the polypeptide also passes as a hairpin through the domains – but in contrast to adaptors it starts and ends in the helical section. Thus it may hint at a deep evolutionary relationship between drug eﬄux assemblies and flagella together with type III secretion structures.

## Models of Full-Pump Assembly and the Respective Role of PAPs in them

While structures of isolated components of the tripartite pumps are available for a number of different species and transporter types, the actual mode of association remains an area of active debate. The RND transporter family was the first group of transporters associated with tripartite pumps for which structures became available, influencing early models of assembly. RND pumps are specific for toxic substrates and largely belong to one of two families: the HME family and the multidrug hydrophobe/amphiphile eﬄux-1 (HAE1) family, both of which have unique PAPs.

RND HAE1 transporters are trimeric assemblies, with each protomer consisting of a transmembrane domain containing 12 transmembrane α-helices and characteristic two large hydrophilic loops that comprise the substrate-binding porter (or pore) domain and the OMF-coupling docking domain (Murakami et al., 2002). The HME pumps have a very similar trimeric assembly (Long et al., 2010), while the general protomer architecture is also shared with SecDF family as well as with the mycobacterial MmpL family of transporters (Tsukazaki et al., 2011; Varela et al., 2012).

### Deep Interpenetration Models

As soon as the AcrB structure became available it was speculated that TolC and AcrB might come into direct contact (Murakami et al., 2002), based on the apparent spatial compatibility of their apex regions. When the first OprM structures became available this idea was further reinforced by Akama et al. (2004), who pointed out the complementarity of the hydrophobic residues present in RND transporters and OMFs. Such direct interaction has been unequivocally demonstrated by *in vivo* crosslinking by Tamura et al. (2005). As mentioned before, the initial idea of PAP function ascribed them membrane-fusion protein like qualities, and suggested that they literally bring the two membranes together (Dinh et al., 1994). In prescient analysis, Johnson and Church (1999) dismissed the fusion protein connection, and suggested for the first time not only the organization of tandem repeats of the TolC-family, but also the potential for the formation of helical bundles between the OMPs and adaptor proteins to stabilize the complete assembly.

Taking into account the then-available MexA structures and this suggestion, Akama et al. (2004), Fernandez-Recio et al. (2004), and Higgins et al. (2004a) proposed the first fully assembled models of the tripartite pump. These models all featured deep interpenetration between the helical hairpin of the PAP and the coiled-coil domain of OMF, but differed wildly in terms of stoichiometry, presenting respectively 3:9:3, 3:6:3, and 3:3:3 options, although Akama et al. (2004) even suggested that up to 12 PAP protomers could be accommodated.

The 3:3:3 model of Fernandez-Recio et al. (2004), featuring a direct interaction between the RND transporter and TolC, has become one of the most popular models of pump assembly and provided the foundation for a number of other models (e.g., Symmons et al., 2009) sharing the same lateral inter-helical bundling between the PAP and OMF, collectively referred to here as “deep-interpenetration” models. These models (for example the AcrAB-TolC model of Figure 5A) are supported by direct evidence from cross-linking studies and a number of gain-of-function analyses, which will be discussed in detail below.

The debate on the stoichiometry of the pumps is still not fully settled. However, following the description of MacA hexameric organization in isolation (Yum et al., 2009); the CusBA crystal structure solution demonstrating a trimer of dimers of CusB (Su et al., 2011); and the direct crosslinking of the PAP hairpins to both grooves of the OMF (Janganan et al., 2011a), the 3:6:3 models have come to dominate the field. Furthermore, the existence of fused dimeric PAPs such as DSY0927 from *Desulfitobacterium hafniense* (Zgurskaya et al., 2009); existence of MDR pumps with multiple PAPs such as TriABC (Mima et al., 2007) as well as functional complementation using fused dimeric AcrA constructs (Xu et al., 2011a) strongly support the idea of a trimer of PAP dimers as the most likely functional assembly.

### Tip to Tip Models of Assembly

Higgins et al. (2004a) were the first to propose lack of direct interaction between the RND transporter and TolC, still maintaining a deep interpenetration model. This idea had a dramatic makeover with the determination of the MacA structure, which was used for a radically new model of interaction (Yum et al., 2009).

The crystal structure of MacA shows the same general configuration as other PAPs at the level of the monomer (Yum et al., 2009). However, due to crystal packing it forms a hexameric tube-like structure, which the authors proposed to be the functional quaternary structure and to be maintained through interactions between the β-barrel domains. As the tube formed from the hairpins was approximately the same diameter as the α-barrel of TolC, they hypothesized that the α-barrels of these oligomeric assemblies may sit one atop the other to form a continuous channel. Following the structure being solved, a new conserved motif was identified at the tip region of the PAP hairpin – the ‘RLS motif’ – which was proposed to be common and essential (Xu et al., 2010). This RLS motif has been studied in various PAPs, with most of the mutations affecting it reported to abolish function and binding of PAP to OMF (Kim et al., 2010; Xu et al., 2010, 2011b; Lee et al., 2012; Song et al., 2014).

The next important advance came when the structure of CusB in isolation and as part of the CusBA complex were resolved in quick succession (Su et al., 2009, 2011), revealing for the first time a binary PAP-RND transporter complex, which presented a 2:1 PAP:transporter stoichiometry. Despite the marked difference of its hairpin domains from those of canonical adaptors, CusB was found to form a ring atop the CusA transporter, with an aperture too narrow to accommodate its cognate OMF CusC, which is structurally very similar to TolC. Extrapolating the structure of a complex of multidrug RND transporter (such as AcrB or MexB) with its associated PAP that has a prominent α-helical domain from that of the CusBA complex reinforced the idea that in such RND-based pumps the OMF does not contact the transporter and may interact with the PAPs in a tip-to-tip fashion. However, while CusB forms a hexameric ring atop CusA, the helices of CusB-hairpins appear to be folded away from the CusC OMF (Su et al., 2011).

**FIGURE 6:**
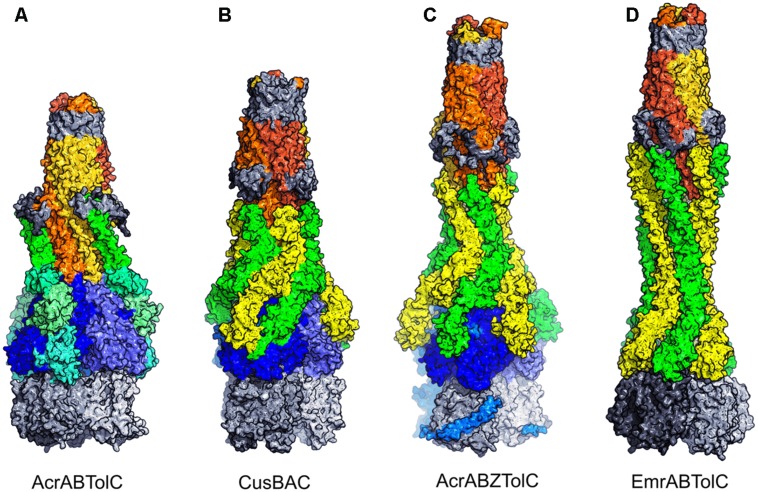
**Assembled models of pumps**. OMF subunits are shown as gold or orange shades except for the beta-barrel in the outer membrane and the equatorial domain, which are in gray. AcrB and CusA trimeric RND transporters are blue for its periplasmic domains, gray for its membrane domains. EmrA MFS transporter is in gray (a dimer is shown for size comparison). Adaptors are in shades of green or yellow around the OMF. **(A)** AcrABTolC model based on site specific cross-linking data (Symmons et al., 2009). Domains of the AcrB are distinguished by green shades. **(B)** CusBAC model based on the docking in Su et al. (2011). This is based on the maximum interpenetration without alteration in the CusB hairpin domains. Closer approach of the CusC and CusA is possible after re-alignment of the adaptor tips. **(C)** AcrABZTolC model based on fitting to single-particle CryoEM envelope (Du et al., 2014). The AcrZ subunit, to which one copy of AcrA was fused, is shown in light-blue. **(D)** EmrABTolC model suggested by the structure of Hinchliffe et al. (2014) and constructed by docking six EmrA subunits based on the arrangement of helices in the TolC trimer (Figure 5B). Further interpenetration would be possible after re-alignment of the adaptor tips.

Crystallographic pursuit of the structure of the complete tripartite complex has been complicated by the transient nature of the inter-component interactions making the isolation of sufficient quantities of monodisperse complexes suitable for crystallographic studies problematic. Thus most of the recent efforts to reconstitute the full complex for structural studies have focused on single particle reconstructions, which required engineering of the components of the complex for increased stability. This approach achieved a major breakthrough by visualizing a complete assembly for the first time, based on cryo-electron microscopy reconstruction, which appears to support a model resembling the prototypical tip-to-tip yet also displaying some limited interpenetration between the tip regions of the PAPs to OMF (Du et al., 2014; Figure 6). At the same time, a negative stain EM reconstruction claimed that a canonical tip-to-tip interaction may take place (Kim et al., 2015).

## Functional and Biophysical Evidence Supporting Different Modes of Assembly

While providing crucial new insights, the recent EM studies used heavily engineered chimeric protein assemblies shown to have only limited functional activity (Du et al., 2014). An earlier EM-tomography study using non-modified MexA–MexB proteins reconstituted into membranes was unable to distinguish between tip-to-tip and deep-interpenetration models (Trépout et al., 2010). These observations call into question whether the conformations being stabilized in these assemblies represent a functional state of the activated pump or perhaps an inactive intermediate. While the question of the architecture of the functional assembly awaits its final solution we systematize the available evidence in the context of each of the models, focusing on the OMF-PAP interaction.

The two different models of OMF-PAP interaction predict dramatically different binding interfaces. In the deep-interpenetration model there are large helical bundling interfaces. This creates two degenerate interfaces, which closely resemble each other, but are not identical. In some systems, such as triclosan pump TricABC-OpmH, each of the grooves is occupied by separate PAPs (Mima et al., 2007), but in the majority of cases a single PAP seals the assembly. This introduces a requirement for some sequence-tolerance of the side of the PAP hairpin, which has to bind two slightly different grooves on the OMF. At the same time, the extended nature of the interface would be more tolerant to single substitutions and some level of promiscuity is expected between related protein pairs. Indeed, a number of studies have reported that the non-cognate OMFs and PAPs assemble, although not always into functional complexes (Bokma et al., 2006; Stegmeier et al., 2006; Vediyappan et al., 2006; Krishnamoorthy et al., 2008; Yoshihara et al., 2009). The hypothesis that the hairpin of the PAP requires a specific interaction with some residues on the surface of the OMF to unlock the “gates” logically expects a limited number of “discriminator” residues to be present to allow differentiation between productive and non-productive complexes (Bavro et al., 2008). Such interpretation is consistent with the study by Stegmeier et al. (2006), which demonstrated a clear separation between interaction and functionality, as WT AcrA could be cross-linked to both TolC and OprM via the DSP reagent, which has a 12 Å spacer arm, but could only confer drug resistance when used with TolC. Similarly, Bokma et al. (2006) also distinguished functional activity from association, as MexA and TolC could be cross-linked whilst being unable to form a functional complex.

While the non-cognate hairpins of the PAPs may not function *ad hoc* due to incompatibility with these residues, one can also expect that the incompatibility in such a scenario could be overcome by adaptation of the interface, e.g., via mutation of these key discriminator residues (Bokma et al., 2006; Vediyappan et al., 2006). To the contrary, the very limited interface of the tip-to-tip models should be much more sensitive to single substitutions specifically at the tip regions of the PAP and the OMF, and as these are expected to be the only determinants of specificity as well as binding affinity, the compensatory mutations would be expected to also map to the same region.

### Evidence from Cross-Reactivity

The concept of simultaneously maintaining both specificity and promiscuity may be thought of as a suited key system, in which the locks are analogous to OMFs and keys analogous to PAPs, whereby locks and keys can be suited to different security levels. Using the same analogy, a functional key fits, turns and unlocks the gate, while a number of similarly shaped keys would only fit the keyhole [e.g., the examples provided by Stegmeier et al. (2006) and Bokma et al. (2006)].

A good illustration for the concept is provided by the existence, within a single species, of PAPs that have multiple cognate OMFs, such as MdsA of *Salmonella*, which can use both MdsC and TolC (Horiyama et al., 2010; Song et al., 2014). In such an analogy MdsA is a more universal key than AcrA, opening more locks; MdsC here would appear to be a more secure lock than TolC, being opened only by one key rather than by many. This can easily be explained using the idea of extended interfaces with discriminator residues. Here, TolC and MdsC would have some discriminator(s) in common; however, the MdsC would have extra, which can only be recognized by MdsA.

The keys analogy would also predict that in some cases there is an odd chance that an OMF may function with a non-cognate PAP from a different species. An example of this is VceAB of *Vibrio*, which pairs with TolC in AcrAB-deficient *E. coli* (Vediyappan et al., 2006). As the reverse is not true (AcrAB cannot function with VceC), VceC could be likened to MdsC, as possessing a higher level of security than TolC, likely due to an extra set of discriminator residues.

A clear demonstration of the importance of the hairpin for the selection of partners can be obtained from domain swap experiments. If a PAP hairpin contains the entire lock-fitting features of a key, then hairpin swapping would change the OMF-binding profile of one PAP to that of another. A study by Stegmeier et al. (2006), which analyzed MexA hairpins grafted onto AcrA, demonstrated that such chimeras can cause gain of function with a non-cognate OMF, but do not necessarily cause loss of function with the cognate OMF. In the case of a stringent fit, one may expect that MexA should also be capable of at least partially functioning with TolC, as AcrA(MexA-hairpin) can. It is therefore surprising that MexAB cannot function with TolC unless directed evolution is used (Bokma et al., 2006), hinting that additional levels of compatibility checks may be in place.

### Evidence from Adaptive Mutagenesis

Since non-cognate PAPs present imperfect keys, directed evolution could help identify discriminator residues. However, the distribution of these gain-of-function mutants would be expected to be markedly different under the different models of assembly. In the report from Bokma et al. (2006), several mutations required to adapt TolC to MexAB occurred in the β-barrel and are difficult to visualize as interacting with any other component of the eﬄux machinery in either model. However, the study also found a number of mutations in the α-helical regions of the OMF both at the tip and high up the coiled-coil domain, consistent with deep interpenetration. An alternative explanation for the gain-of-function may be that the mutations cause the channel to become leaky, such that they do not require opening by the PAP. Similarly, gain of function mutations in VceC allowing it to function with AcrAB are spread around the lower portion of the α-barrel (Vediyappan et al., 2006), but are not confined to the tip. One (V445E) affects the hydrophobic gate of VceC in the equivalent position to D374 in TolC (Koronakis et al., 2000; Federici et al., 2005), and would likely introduce a similar acidic-residue ring. The existence of compensatory mutations far away from the tip region is difficult to reconcile with the tip-to-tip models, as the functional interaction, and hence its loss, is supposed to be restricted to the limited tip region. Hence, a gain of function would be expected to arise at the same interface. In stark contrast the majority of the Vediyappan et al. (2006) mutations map to the inside of the channel, ruling out their role in direct engagement with the PAP.

### Evidence from Compensatory Mutations

Similar to directed evolution of non-cognate OMF-PAP pairs, the mapping and characterization of the gain of function mutations that compensate defects on either of the components of the pump complex provide powerful tools for studying the mode of their interaction. Weeks et al. (2010) reported on the effects of extensive mutagenesis of the periplasmic turn connecting the first two helices of the TolC channel, which, in the strict tip-to-tip models of interaction comprises almost half of the expected docking site for the PAP. Due to the very limited size of the tip, one might expect the mutagenesis to cause severe disruption of the interaction, however, this isn’t the case. Even when the signature sequence GLVA was substituted to a poly-Ala the OMF retained wild-type functionality, and only mutation of all four positions to AGSG caused loss of function. This insensitivity implies either extensive structural redundancy or potentially a different mode of interaction between the OMF and the PAP taking place. That conclusion is further reinforced by the isolation of AcrA suppressors of the AGSG, which were shown to dilate the TolC aperture in an AcrB-dependent manner. Furthermore, this did not require energy input from AcrB, as the induction of leakiness was also present in AcrB D407 mutant, lacking functional proton coupling (Weeks et al., 2010). Interestingly, out of the six compensatory mutations isolated, only a single one, T111P, was located at the hairpin.

The location of multiple compensatory PAP mutations at the level of the RND-transporter suggests that the rescue of eﬄux function may occur via stabilization of the PAP-transporter interaction, leading to extended lifetime of the eﬄux complex. This is consistent with the observation that AcrA-recruitment of proteinase sensitive TolC mutant P246R/S350C into complexes protects it from degradation (Gerken and Misra, 2004; Weeks et al., 2014). Similar observations have been made by Nehme and Poole (2007), who reported that RND transporter mutation (MexB G220S), which caused a loss of transporter-PAP association and resulted in drug sensitivity, was compensated by mutations in the α-barrel of the OMF promoting increased stability of OMF-PAP association. Mutation at the tip of MexA α-hairpin (V129M) compromised the *in vivo* interaction with OprM resulting in drug hypersensitivity, which may hint at a tip-to-tip interaction. However, that phenotype was restored by the T198I and F439I substitutions 5 helical turns up the α-barrel of OprM, consistent with the hairpin domain mediating MexA binding to this region of OprM in a lateral fashion (Nehme and Poole, 2007). Furthermore, the association between the mutant MexA and OprM was not affected, indicating that impacted gating, rather than disrupted complex formation, caused the observed eﬄux defects.

### Cross-Linking Data

Usage of heterobifunctional cross-linkers with different spacer lengths achieved *in vivo* cross-linking of PAPs to OMFs (Lobedanz et al., 2007). In these studies, cysteine residues introduced along the entire length of the N-terminal helix of the AcrA hairpin could crosslink to TolC, when using a 6.8 Å linker arm. This suggests that the residue eight helical turns from the PAP tip must lie less than 7 Å from TolC. The residue one helical turn further from the tip could only be cross-linked to TolC with the longer (15.6 Å) linker arm. These results suggest a deep interpenetration of at least six helical turns. Introduction of a cysteine in TolC, six helical turns from the helical tip, could also be cross-linked to AcrA via the short-spacer linker. At the same time a TolC D121C mutation, seven helical turns from the tip, could not be cross-linked with either linker. Given that a D121N mutation was identified as an adapting mutation that enables TolC to function with MexAB (Bokma et al., 2006), a charged residue may be involved in maintaining the PAP association.

### Evidence from Direct-Residue Interactions

Interpretation of the data generated by heterobifunctional cross-linking is complicated by the uncertainty introduced by the length of the spacers and the involvement of large side-chains, e.g., Lys and Arg. It is more difficult to refute results from direct spontaneous Cys–Cys cross-linking and functional complementation. One example of a direct interaction between the OMF and the PAP was described by Bavro et al. (2008) in the case of the K383 (TolC)-D149 (AcrA) functional pair. Mutation of each of the residues in isolation caused hypersensitivity to the AcrB substrate novobiocin, presumably due to abolition of the OMF-PAP association. The functional activity could be restored when the reciprocal mutations were introduced into the respective proteins, suggesting a direct interaction between the two. Mutation of the equivalent residue to K383 in the Neisserial ortholog MtrE (E434) similarly causes hypersensitivity to substrate drugs, but also makes the cells sensitive to the influx-dependent vancomycin, indicating that the mutation causes the OMF channel to become leaky (Janganan et al., 2011b). Importantly, vancomycin hypersensitivity was only observed when the OMF was co-expressed with the PAP, suggesting that their interaction is required to provoke channel opening (Janganan et al., 2011a, 2013).

Several other MtrE mutations affecting eﬄux have been identified, all of which map to the surface of its α-barrel, up to eight helical turns from its periplasmic tip-region. The loss of eﬄux function was not related to the failure of association, as binary OMF-PAP complex formation was not affected, as demonstrated by isothermal calorimetry (ITC) and pull-down assays (Janganan et al., 2011b). Furthermore, introduction of MtrC E149C and MtrE K390C resulted in formation of intermolecular Cys–Cys bridging *in vivo*, locking the OMF channel in an open conformation thus causing increased vancomycin sensitivity (Janganan et al., 2011a).

These results, combined with the similar cross-linking studies of AcrAB (Symmons et al., 2009), served as the principle source of the refined deep-interpenetration model of pump assembly.

### Evidence from Structural Biology Studies

Unlike the deep-interpenetration model, which was primarily derived from *in vivo* functional and cross-linking assays, the main support for the tip-to-tip model came from *in vitro* structural studies of isolated components. While CusBA crystallographic complex is sometimes considered as supportive of tip-to-tip assembly due to the narrow aperture of the ring of the PAPs which may imply that there is no direct contact between the transporter and the OMF, the organization of the CusB hexamer is rather different from that in the MacA structure (Yum et al., 2009; Su et al., 2011, 2012). It is in fact a trimer of dimers, and the hairpins of the PAP in the case of CusB are pointing away from the center, without participating in tubular formation. Also, the very size of the CusB hairpin dictates a necessary adjustment of the OMF-interaction distance for a productive complex to form in a tip-to-tip model as evidenced on Figure 6.

Apart from the crystal structures of MacA and CusBA, the majority of these studies included different degrees of usage of chimeric proteins. Chimeric constructs of *Actinobacillus actinomycetemcomitans* (*Aa*) MacA on which the tip region was replaced by the tip regions of the TolC α-barrel have been analyzed for structural formation with wild-type *E. coli* MacA by electron microscopy, and showed dumbbell-shaped structures with a central bulge (Xu et al., 2011b). Similar studies, replacing the hairpin tip of *E. coli* MacA with that of MexA or AcrA and the hairpin tip of *Aa*MacA with the tip regions of the OprM or TolC α-barrel showed the same bulged dumbbell-shaped structures (Xu et al., 2011a, 2012). In all of these studies the bulges in the structures were modeled as an intermeshing of the tip regions of the two proteins, with the OMF aperture fully opened. The MexA-OprM docking model suggested possible interacting positions, with the RLS motif formed of R119, L123 and S130 of the MexA proposed to interact with the OprM backbone carbonyl groups, V201/V408, and S138 of OprM, respectively, with additional hydrophobic support from MexA L122 with OprM V199/T406 (Xu et al., 2012).

The recent electron microscopy studies of complete assemblies have provided the most compelling support for the tip-to-tip interactions to date (Du et al., 2014; Kim et al., 2015; Figure 6). It is notable that the two models derived from these EM-reconstructions differ slightly on the level of OMF-PAP interaction. While Kim et al. (2015) have put forward an orthodox tip-to-tip interaction, where only the RLS motif and the turns of the TolC channel seem to interact, the envelope provided by Du et al. (2014) appears to allow for at least partial interpenetration of the OMF and the PAP. Thus this latter model might be able to rationalize at least some of the evidence presented above, and is compatible with the direct disruption of secondary gates by the PAP. However, in common with earlier such models it rules out a direct interaction with the RND-class transporters (Du et al., 2014).

### Evidence from *In Vitro* Binary Interactions between Components

Apart from EM studies, some support for the tip-to-tip interactions comes from recent SPR studies of the *Anabaena* DevBCA ABC-transporter system, the PAP in which is DevB, was reported to require the tip-regions of TolC for binding (Staron et al., 2014). However, surface plasmon resonance (SPR) studies of a number of PAPs as well as TolC, have detected direct interaction of the OMF with the RND transporters which possess large periplasmic domains, independently of the PAP (Tikhonova et al., 2011). The binding is enhanced by low pH, dependent on lipidation and reported to be of nanomolar affinity. Mutations affecting the aperture of the TolC channel by disruption of the primary gates resulted in decreased binding to AcrB and AcrA, implying that the tip regions were indeed specifically engaging under the test conditions (Tikhonova et al., 2011).

Isothermal calorimetry measurements of binding of the PAP MtrC and OMF MtrE showed that the PAP hairpins in isolation bind the MtrE channel with around fivefold higher affinity than the full-length MtrC. This could be increased to 100-fold (13 mM) when a leaky E434K OMF mutant is used as a partner (Janganan et al., 2011b).

### Evidence from RLS Conservation and Diversity of the PAP Hairpins

Although the proposed RLS motif seems to be widely conserved between different pump systems (Kim et al., 2010; Xu et al., 2010), this conservation is not absolute, and deviation from the canonical sequence has been reported, e.g., in the HlyD family of PAPs (Lee et al., 2012). Some other TolC-binding PAPs in *E. coli* do not seem to possess identifiable RLS sequence altogether – e.g., CvaA (Hwang et al., 1997), suggesting that an alternative interaction can take place at least in some instances. The EM analysis of chimeric constructs, implies that at least part of the interaction is backbone mediated (Xu et al., 2011a, 2012), which seemingly contradicts the strict requirement for RLS conservation.

Perhaps the biggest challenge for the tip-to-tip model is the existence of eﬄux assemblies lacking not just the RLS motif but the entire hairpin, such as the *Borrelia burgdorferi* BesA, which is associated with the RND transporter BesB (Bunikis et al., 2008; Greene et al., 2013). While such an assembly may still be reconciled with a deep-interpenetration model where the RND participates in direct binding with the OMP, it is fully incompatible with the current tip-to-tip models. Furthermore, consistent with the hypothesis that one of the main roles of the PAP hairpin domain is to unlock the secondary gates of the OMF, uniquely the TolC homologue BesC associated with this hairpin-less eﬄux system has a disrupted gate system.

### Evidence of Equatorial Domain Involvement

One of the adapted lines from Bokma et al. (2006) that contained two mutations in TolC that greatly increased its functionality with MexA may have increased the propensity for cross-linking. This double mutation increased antibiotic resistance in an additive fashion compared to individual mutations, though one (V198D, in the equatorial domain) had a greater effect than the other (Q142R, at the tip region), suggesting a role for the equatorial domain in determining specificity. It was shortly after determination of the TolC structure (Koronakis et al., 2000), that evidence first arose suggesting the equatorial domain may be involved in OMF function (Yamanaka et al., 2001, 2002). These equatorial domain mutations affected function without affecting stability or folding of TolC, as shown by cross-linking and immunoblotting. Evidence for the significance of the equatorial domain has also been found in the OMF AatA, where positions F381, L382 and L383 have been shown as essential for Aap secretion (Iwashita et al., 2006). These positions mapped to the equatorial domain as based on the homology model of AatA (Nishi et al., 2003). It is also notable that, pairing with a PAP lacking a hairpin domain altogether, BesC not only lacks primary gates but the C-terminal domain is also truncated (Bunikis et al., 2008; Greene et al., 2013). The significance of the equatorial domain has also been shown in the OMF OprM, in which C-terminal truncation impairs the ability of OprM and VceAB to form a functional complex (Bai et al., 2010, 2014).

### Evidence from TolC-AcrB Direct Interactions

As both AcrB and TolC protrude into the periplasm from the inner and outer membrane respectively, Murakami et al. (2002) suggested that they directly dock with each other at their periplasmic tips, which have remarkably similar spatial-dimensions and structural complementarity. The suggested TolC-docking site of AcrB covers part of the “TolC-docking domain,” and features two β-hairpin extensions, while TolC contributes two homologous helical turns. This idea was reinforced by direct *in vivo* Cys–Cys cross-linking of the periplasmic turns of the TolC with these β-hairpins (Tamura et al., 2005), even in the absence of AcrA. Consistent with Tamura’s findings, AcrA-AcrB association was found to be independent of the AcrB β-hairpins, however, TolC is lost from the complex when the β-hairpins of the tip of the periplasmic domain of AcrB are deleted (Weeks et al., 2014). Similar to Tamura, earlier reports using cross-linking via DSP showed that the AcrB-TolC proximity was independent of AcrA, although the authors did not detect a direct AcrB-TolC interaction when using isothermal titration calorimetry (Touzé et al., 2004).

### Additional Lines of Evidence

The demonstration that tandem fusions of AcrA provide functional complementation to AcrA deletion, suggesting that PAP dimers may be the functional units for complex assembly is often taken as supporting the tip-to-tip model (Xu et al., 2011a). However, it can equally be accommodated into deep-interpenetration models. The existence of the functional dimeric unit of the PAP has been confirmed by SPR (Tikhonova et al., 2011).

The remaining evidence for how the complex assembles, while strongly favoring the deep-interpenetration model does not, however, disprove the tip-to-tip model entirely. It is still plausible that this model may represent an intermediate step in binding, as initially suggested by the creators of the tip-to-tip model (Yum et al., 2009).

## Functional Roles of PAPs Beyond Structural Assembly

### Energy Independence of Assembly

Effective eﬄux is dependent on energy provision by the transporters, and could be abrogated by proton gradient decouplers such as CCCP (carbonyl cyanide 3-chlorophenylhydrazone) and/or non-hydrolysable ATP-analogs. Given this, it has been anticipated that energy is also required for the formation of the complex, and likely conformational changes in the transporter are relayed to the OMF channel, *via* the PAP, causing its opening. However, several studies have provided evidence that this may not be the case. Several binary interaction studies in the absence of active energy sources have been able to demonstrate successful PAP-OMF association *in vitro*, including EM-studies of reconstituted complexes (Trépout et al., 2010), ITC (Janganan et al., 2011b), and SPR (Tikhonova et al., 2011; Lu and Zgurskaya, 2013). Some early studies on the Type I secretion system HlyBCD have suggested that the assembly of the complex is nucleotide-independent, while the secretion of the HlyA cargo required HlyB-mediated ATP hydrolysis (Thanabalu et al., 1998).

Crucial evidence came from studying the RND MtrCDE system in *Neisseria*, where the opening of the OMF channel was demonstrated to be dependent on the functional interaction with the PAP (Janganan et al., 2013). This interaction was found to cause the E434K mutant of the MtrE to become vancomycin sensitive, but only when co-expressed with full-length cognate PAP. This interaction was transporter independent, and did not require energy. Furthermore, when a transporter mutant lacking a functional proton-relay was introduced the vancomycin sensitivity was greatly diminished, while the sensitivity to drugs translocated by AcrB remained the same, suggesting that a full, but non-productive eﬄux complex is assembled, sequestering the otherwise leaky channels. Similar effects were reported for AcrAB-TolC by Weeks et al. (2010).

These results suggest that energy is required for the eﬄux and disassembly of the pump complex, but not for the association between its components. This provides rationale for future design of peptidomimetic drugs to target the assembly interface of eﬄux complexes at the level of PAP association. Similar approaches have been shown to be effective in targeting the LptD assembly of *Pseudomonas* (Srinivas et al., 2010).

### Active Participation of Adaptor Proteins in Transport Activity of the IMPs

The participation of the PAPs in transport activity may broadly be split into two major actions – namely affecting energy generation and transduction, and participation in cargo selection and presentation to the transporter. The active role of PAPs in regulating the transporter energy cycles was initially demonstrated for the ABC transporters. The PAP MacA has been shown to be critical for ATPase activity of MacB (Tikhonova et al., 2007; Lin et al., 2009; Modali and Zgurskaya, 2011). Modali and Zgurskaya (2011) further narrowed down the region responsible for this activation to the MPD, and proposed that the MacA adaptor protein promotes the transporter MacB transition to a closed ATP-bound state, similar to the structurally unrelated periplasmic solute binding proteins, such as TroA (Deka et al., 1999).

The role of PAPs in activation of proton-motive force driven transporters is less well explored. This is mainly due to the difficulties in reconstituting active systems utilizing proton-motive force. However, it is emerging that PAPs play a significant role in stimulation of the eﬄux activity and consumption of the gradient as exemplified by the reconstitution of MexA–MexB into liposomes (Verchère et al., 2012). MexA dramatically increased the activity of MexB only when the substrate was also present, confirming and expanding the results of earlier AcrA–AcrB liposome reconstitution assays (Zgurskaya and Nikaido, 1999). These results invite the exciting speculation that one of the roles of PAPs could be to serve as checkpoints for successful drug loading into the transporter, to prevent unproductive cycling without cargo that may deplete the proton gradient.

In order to effectively fulfill such checkpoint function, the PAP may be expected to participate in cargo binding and selection, and there is mounting evidence from different systems to support such a hypothesis. One early report described substrate-induced conformational changes in the MFS-associated EmrA from Trp-fluorescence analysis (Borges-Walmsley et al., 2003).

#### Heavy Metal Eﬄux

The heavy metal eﬄux (HME) pumps have been instrumental for establishing the active role of the PAPs in the transport process. De Angelis et al. (2010) demonstrated that the PAP ZneB of the ZneCAB heavy-metal eﬄux system from *Cupriavidus metallidurans* specifically binds Zn^2+^ ions in the interface between the β-barrel and MPD domains. Binding is associated with a significant conformational change and on this basis it was suggested that the PAP may play an active role in the presentation of the substrate to the transporter ZneA. Similar action has since been confirmed in the Cu(I)/Ag(I) eﬄux pump CusCFBA which is composed of the OMF CusC, the RND-transporter CusA, metallochaperone CusF, and the PAP CusB. CusF and CusB have been shown by NMR spectroscopy to freely exchange Ag(I) and Cu(I) toward equilibrium in highly specific protein–protein interactions (Bagai et al., 2008; Mealman et al., 2011). Similar organization has been found in the PAP SilB from *Cupriavidus metallidurans* CH34 which has a C-terminal-extension domain homologous to CusF (Bersch et al., 2011).

Metal co-ordination appears to be accomplished by methionine clusters, in both the chaperones and the transporter (e.g., CusA) as identified by X-ray crystallography and NMR and by mass-spectrometry and X-ray absorption spectroscopy (Su et al., 2011; Mealman et al., 2012), creating an ion-transport relay. The latter study also demonstrated that the N-terminal 61 residues of CusB are sufficient to bind metal and provide partial metal resistance *in vivo*. It has also been shown that the N-terminal domain acquires the metal from the metallochaperone (CusF) and is able to pass it on to the transporter (Mealman et al., 2012; Chacon et al., 2014). In that study, CusB was found to directly activate the CusA pump.

#### RND Eﬄux Pumps

The involvement of the PAPs in the cargo selectivity in the RND multidrug eﬄux pumps is less studied, but some indication of their role could be found from studies of non-cognate PAP complementation. Change of the substrate profile brought by the PAP change was clearly demonstrated by the complementation analysis of AcrA interactions with MexB (Krishnamoorthy et al., 2008). In this system AcrA was able to provide near wild-type resistance to SDS, and partial to novobiocin and ethidium bromide, while nalidixic acid, lincomycin, and erythromycin proved highly toxic, suggesting that the change of PAP resulted in a shift of substrate specificity of the pump.

### Interactions within the Membrane

As mentioned previously, some adaptor proteins contain N-terminal membrane spanning domains, and these have been suggested to interact within the membrane with their cognate transporters (Tikhonova et al., 2007). This is likely the prime way of communication between transporters that lack any periplasmic protrusions and are fully submerged in the membrane, such as the canonical ABC transporters and MFS transporters. In HlyD, a Δ-N45 construct lacking the N-terminal cytoplasmic helix failed to recruit TolC or activate the HlyB ATPase, suggesting that a transmembrane communication takes place (Balakrishnan et al., 2001).

### Importance of the C-Terminal Domain of the PAP

Elkins and Nikaido (2003) showed that the C-terminal part of the PAP plays a role in the recognition of the transporter. The region identified encompasses the majority of the MPD, consistent with that identified by Ge et al. (2009), showed that a single G363C substitution in the MPD dramatically impairs the multidrug eﬄux activity of AcrAB-TolC. The importance of the MPD has also been noted in the ABC-transporter associated MacA, where substitutions in the MPD affected LPS binding as well as general activity of the pump, including macrolide eﬄux (Lu and Zgurskaya, 2013). One interesting observation from earlier work (Tikhonova et al., 2002), showed that a small region of the RND transporter was crucial for binding with the PAP. Mapping this region to the available binary complex of CusBA (Su et al., 2011), shows that the equivalent sequence in the CusA overlaps with its docking site for the CusB MPD. Interestingly, the bound protomers of CusB display significant conformational discrepancy at their respective binding sites. The corresponding region would also be close to suggested drug-acquisition sites in AcrB (Pos, 2009). This raises the intriguing speculation that the MPDs may be actively sensing the state of the transporter, translating it into communicable conformational change.

It is notable, that MPDs appear exclusively in PAPs associated with RND- and ABC-transporters that feature prominent periplasmic domains. As these classes of transporters are also known to acquire their eﬄux substrates from the periplasmic space or the outer leaflet of the cytoplasmic membrane, we propose that the role of the MPDs in these systems may be associated with active cargo presentation and regulation of energy-coupling of the transport cycling.

ATPase activation of the transporter and active involvement of the adaptor in cargo binding and presentation is not limited to transporters with large periplasmic domains. Direct binding of cargo to HlyD has been reported (Balakrishnan et al., 2001). Substrate binding was not dependent on the N-terminal helical domain, as HlyD was still able to associate with both substrate and TolC. However, the substrate transport was impaired, suggesting that this region may play an active role in assembly and stimulation of the ATPase activity of the HlyB transporter. The recruitment of TolC to preassembled HlyBD was promoted by cargo binding (Thanabalu et al., 1998; Benabdelhak et al., 2003). Such recruitment may result from conformation changes in the PAP, as suggested from crystallographic and molecular dynamics studies (Mikolosko et al., 2006; Vaccaro et al., 2006; Wang et al., 2012), where the PAP hairpin flexes relative to other domains in a pH-dependent fashion (Ip et al., 2003), which may mimic *in vivo* functional binding to cargo and/or transporter. Furthermore, it has been reported that mutations in the PAP HlyD affected folding of the substrate (Pimenta et al., 2005). One such mutation maps within the hairpin domain, highlighting a role of hairpins in folding, perhaps by creation of a “foldase” cage, which may explain the presence of these domains in Gram-positive organisms.

## PAPs in Gram-Positive Organisms

The very existence of PAPs in Gram-positive organisms suggests that their roles must be much more diverse than just bridging between the transporter and OMF. Based on the same logic it may also be expected that the ones present would be lacking α-hairpin domains. This has proven not to be the case, however, and genome analysis studies have revealed a number of PAPs are indeed present in Gram-positive organisms (Zgurskaya et al., 2009), contrary to the early expectations (Dinh et al., 1994).

While in some cases it is difficult to establish functionality of these genes, which may have been acquired via a lateral gene transfer and are dormant in the genome – e.g., in the case of *Enterococcus gallinarum* EGD-AAK12ERE46183.1 which shows up to 82% identity to the MFS-associated EmrA hairpin domain; there are a number of *bona fide* secretion systems in firmicutes that require PAPs for function. ABC associated PAPs similar to HlyD could be readily identified, e.g., MknX from *Bacillus*. Another wide spread system is the mesentericin Y105 secretion pump which is built around the MesD-type ABC transporter (Aucher et al., 2005). The gene encoding this transporter pairs with the *mesE* gene, which appears to encode a PAP resembling HlyD. Some examples include MesE from *Leuconostoc mesenteroides* (Q10419.1), PlnH from *Lactobacillus plantarum* (WP_015379778.1); SppE from *Lactobacillus sakei* (CAA86947.1). The list could be expanded by the related Streptococcal competence protein ComB, as well as the *Enteroccocal* iron ABC transporter (WP_025481776.1), and *Carnobacterium maltaromaticum* CbaC (AAF18149.1).

These PAPs possess extremely large hairpin domains, which is difficult to rationalize if the only function of the hairpin is OMF-transporter bridging. However, as mentioned above it may have a role in folding the nascent polypeptide chain of the cargo. Preservation of the large hairpins in PAPs such as MdtN in *Virgibacillus halodenitrificans* showing high similarity to the PAPs associated with MFS transporters [e.g., EmrX-MdtN-MdtP(OMF) in *E. coli*] is more difficult to explain and requires further studies.

A rather unusual case is presented by the *Bacillus subtilis* YknX (BSU14350), which seems to function with a multi-component ABC-transporter, YknZYW, involved in the resistance to antimicrobial killing factor SdpC (Yoshida et al., 2000; Yamada et al., 2012). This system contains a permease, YknZ, which binds to the transmembrane regulator YknW, while ATPase activity provided by a separately coded YknY powers the full assembly (Zgurskaya et al., 2015). All these permeases appear to belong to the FtsX family (Crawford et al., 2011), which also includes MacB and similarly some of them occur also as gene fusions with the AAA-ATPase (e.g., YknU BSU14320 and YknV BSU14330). It is worth reminding that the FtsX-like transporters (including MacB) seem to possess regulatory domains with the ribokinase-fold with striking similarity to the β-barrel domain of PAPs (see Figure 5). Adding to the similarity with MacAB, the PAP YknX also has a prominent α-hairpin domain. Furthermore, it also contains, so far uniquely amongst the Gram-positive PAPs, a MPD. As we will demonstrate in the final chapter, the presence of certain domains can serve as a reliable diagnostic for the pairing of the PAP with its transporter.

## Transporter Type Determines the Domain Organization of the Associated PAPs

**FIGURE 7:**
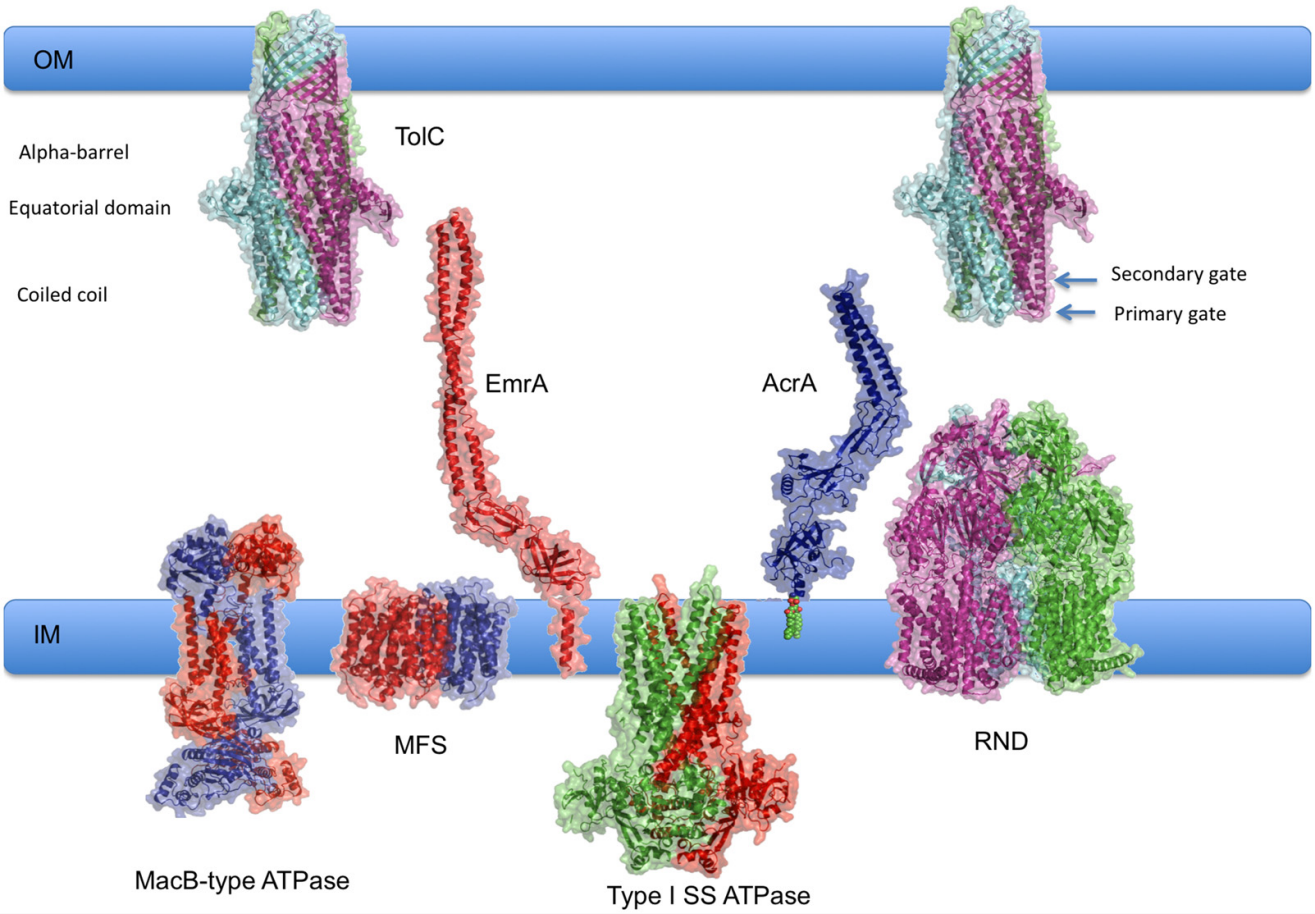
**Schematic representation of the proposed pattern of pairing between PAPs and specific transporters, highlighting domains involved**. We propose that the type of the transporter is strongly linked to the particular architecture of the PAP. This emerging logic allows predicting the type of the transporter based purely on the architecture of the PAP, e.g., membrane proximal domains are only found in PAPs that work with transporters with prominent periplasmic domains, suggesting a role in substrate presentation (see main text for details). In contrast, PAPs which pair with transporters with cytoplasmic cargo access require TM (transmembrane) domains to communicate with the transporter as well as hairpin-extension domains to ensure safe conduit for their cargoes through the periplasm. NBD, nucleotide binding domains.

Our structural analysis of the available PAP-transporter pairs in combination with the examination of the available biochemical evidence, leads us to believe that there is a very clear pattern of structural matching of specific PAP domain combinations to certain transporter types, summarized in Figure 7.

This pairing is far from random and most likely underlies a functional connection between the domains in question. We have identified that MPDs occur without exception in PAPs paired with transporters possessing large periplasmic domains and which are suggested to load their cargo either exclusively or preferentially from the periplasm or the outer leaflet of the inner membrane, such as RND-transporters and MacB-family of ABC transporters. There are two likely explanations for this – one is that due to purely spatial requirements the MPDs are required as “spacers” to prevent displacement of the PAP by the large transporter, which would prevent the PAP from reaching from the inner membrane to the OMF. An alternative and, in light of the increasing amount of functional data, more likely explanation is that the MPDs critically complement the cargo selection and presentation to the transporters.

Similarly, the requirement for effective sealing of the transport conduit in PAP-transporter combinations, such as MFS and ABC-transporters of the Type I Secretion System where the transporter lacks a pronounced periplasmic domain, necessitates an introduction of the recognizable “hairpin extension” subdomain (Hinchliffe et al., 2014). The predicted rigidity of these α-tubular conduits also makes it difficult to imagine major conformational changes being easily communicated through to the OMF channel causing its opening.

Ultimately, none of the evidence provided above proves conclusively either of the models of OMF-PAP association. However, while structural biology seemingly gives us a hint toward the tip-to-tip interaction taking place, the ability of such interactions to provide sufficient energy for stabilization of a large multi-protein assembly, which also undergoes significant conformational changes during its transport cycle, is still called into question, especially in light of the wealth of functional data.

## Conclusion

Recent data have shown the PAPs to be a versatile group of proteins which, far from being passive linkers between the OMF and the energized inner-membrane transporters actively control the tripartite complex assembly. They play an active role in assembly energetics and cargo selection and presentation, while at the same time providing highly specific differentiation between a number of homologous partners to provide high fidelity and specificity of transport.

Our analysis has expanded the current understanding of structural relations of the multiple domains of these highly modular proteins, and revealed some unexpected connections linking them to other secretion systems and transporters. Finally, we have identified a pattern in the domain organization of the PAP families, which underlies their functional association with their cognate transporters.

Summing up the available data also shows that despite these recent advances, the ultimate answer of the complete pump architecture remains elusive.

## Conflict of Interest Statement

The authors declare that the research was conducted in the absence of any commercial or financial relationships that could be construed as a potential conflict of interest.
