# Supplementary material for: Architecture and roles of periplasmic adaptor proteins in tripartite eﬄux assemblies
Source: Front Microbiol. 2015 May 28;6:513. doi: 10.3389/fmicb.2015.00513 (PMC4446572; doi:10.3389/fmicb.2015.00513)

Supplementary Materials:

Figure S1: Structural connections between the beta-barrel domains of the PAPs and flagellar proteins.

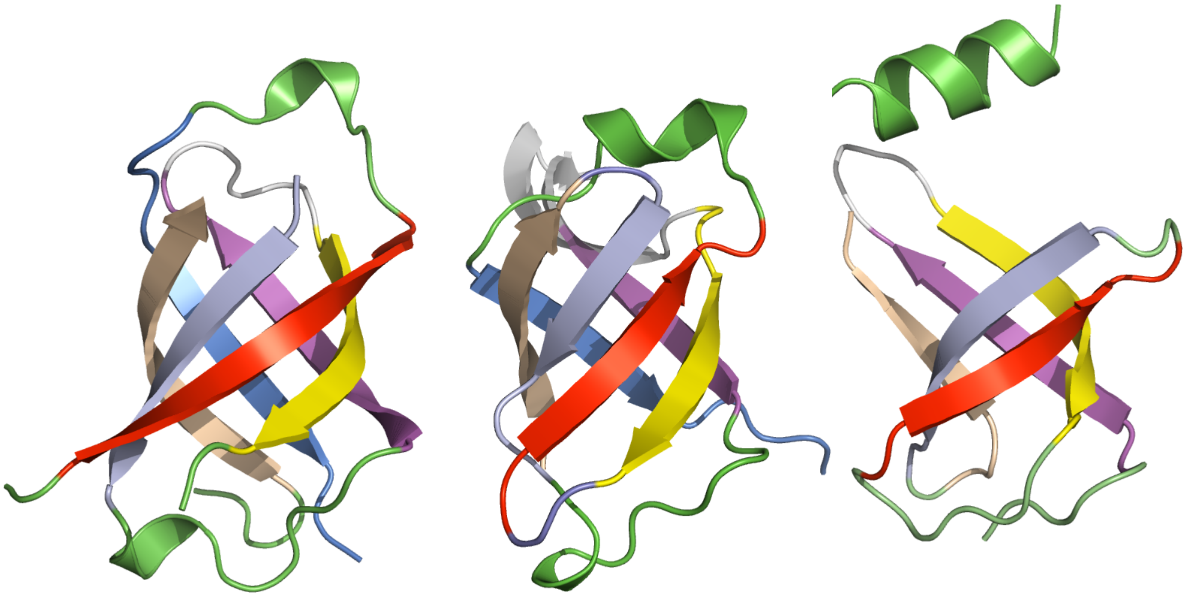

S1A: Comparison of the architecture of the 6-stranded beta-barrel domains of EmrA (left), FlgT (centre) and the "incomplete" 5-stranded barrel of FlgA (3TEE.pdb) (right). Equivalent structural elements highlighted using the same colors.

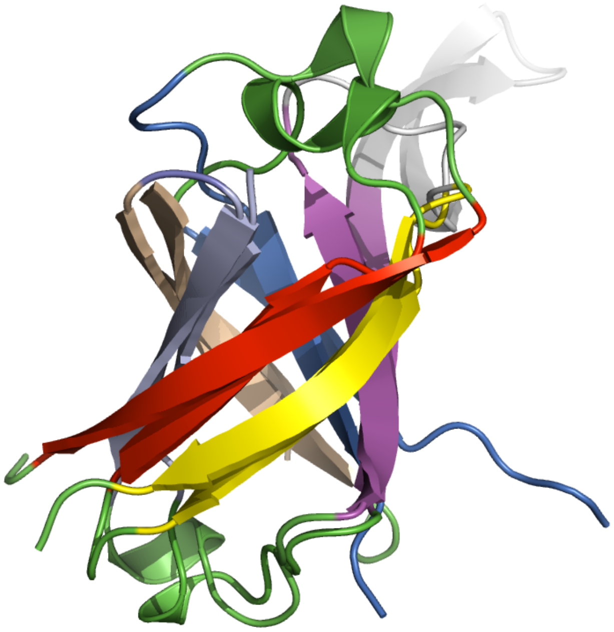

S1B: Overlay of the beta barrel domain of EmrA (4TKO.pdb) and the middle domain of the flagella basal body protein FlgT from *Vibrio* (3W1E.pdb). The two proteins have superpose with an rmsd of 3.9 Angst.

Supplementary Figure 2:
Structural relationships between the beta-barrel and membrane proximal domains (MPDs). Equivalent secondary structural elements are colored equally. Left – a representative beta-barrel domain from EmrA (4TKO.pdb), Right - an MPD domain from MexA (2V4D.pdb).

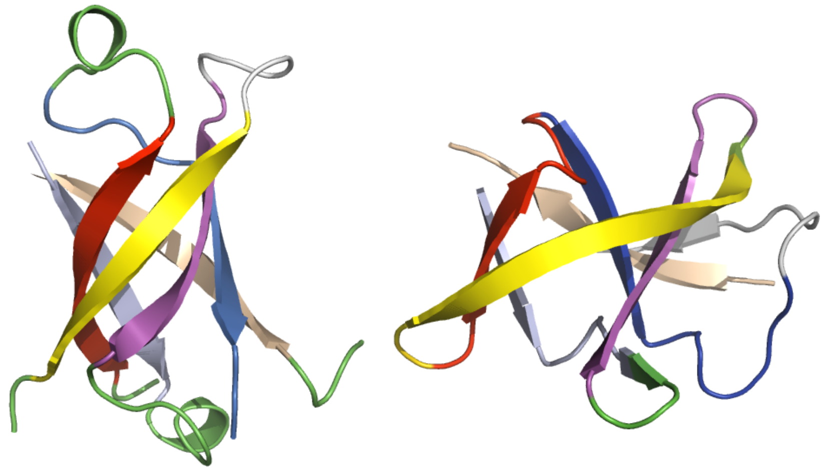

Supplement: Supplementary file 1 [file Presentation_1.PDF]
